# Rational design of biosafe crop resistance to a range of nematodes using RNA interference

**DOI:** 10.1111/pbi.12792

**Published:** 2017-08-22

**Authors:** Hugh Roderick, Peter E. Urwin, Howard J. Atkinson

**Affiliations:** ^1^ Centre for Plant Sciences University of Leeds Leeds UK

**Keywords:** RNA interference, nematode resistance, double‐stranded RNA, transgenic hairy roots, banana, crop

## Abstract

Double‐stranded RNA (dsRNA) molecules targeting two genes have been identified that suppress economically important parasitic nematode species of banana. Proteasomal alpha subunit 4 (*pas‐4*) and Actin‐4 (*act‐4*) were identified from a survey of sequence databases and cloned sequences for genes conserved across four pests of banana, *Radopholus similis*,* Pratylenchus coffeae*,* Meloidogyne incognita* and *Helicotylenchus multicinctus*. These four species were targeted with dsRNAs containing exact 21 nucleotide matches to the conserved regions. Potential off‐target effects were limited by comparison with *Caenorhabditis*,* Drosophila*, rat, rice and *Arabidopsis* genomes. *In vitro act‐4* dsRNA treatment of *R. similis* suppressed target gene expression by 2.3‐fold, nematode locomotion by 66 ± 4% and nematode multiplication on carrot discs by 49 ± 5%. The best transgenic carrot hairy root lines expressing *act‐4* or *pas‐4* dsRNA reduced transcript message abundance of target genes in *R. similis* by 7.9‐fold and fourfold and nematode multiplication by 94 ± 2% and 69 ± 3%, respectively. The same *act‐4* and *pas‐4* lines reduced *P. coffeae* target transcripts by 1.7‐ and twofold and multiplication by 50 ± 6% and 73 ± 8%. Multiplication of *M. incognita* on the *pas‐4* lines was reduced by 97 ± 1% and 99 ± 1% while target transcript abundance was suppressed 4.9‐ and 5.6‐fold. There was no detectable RNAi effect on nontarget nematodes exposed to dsRNAs targeting parasitic nematodes. This work defines a framework for development of a range of nonprotein defences to provide broad resistance to pests and pathogens of crops.

## Introduction

Cooking bananas are an important staple crop for 400 million people in low‐ and middle‐income countries throughout the tropics; in Africa, 70 million people rely on them for more than 25% of their carbohydrates (FAOSTAT, 2014; Ortiz and Vuylsteke, 1996). Annual production of 139 million tons is predominantly by small‐scale farmers to feed their families and for sale at local markets (FAOSTAT 2014). Consequently, the crop is not subject to the same price shocks as other globally traded staples such as rice and wheat (West *et al*., 2014). In tropical regions, where the crop is grown, cooking banana is also capable of feeding more people cheaply per unit area than other staple crops (Frisson and Sharrock, 1999), as such it has an important role to play in ensuring food security in regions of the world that suffer from chronic malnutrition.

Banana is a sterile, genetically homogeneous crop that is severely hampered by several pests and pathogens. In particular, banana plantations can suffer over 50% production losses per crop cycle as a result of plant‐parasitic nematode infections (Norgrove and Hauser, 2014; Roderick *et al*., 2012; Ssango *et al*., 2004). Control of the nematode species complexes of *Radopholus similis*,* Pratylenchus goodeyi*,* P. coffeae*,* Helicotylenchus multicinctus* and *Meloidogyne* spp. is required across many banana‐growing regions (Bridge *et al*., 1995; Gowen *et al*., 2005; Kashaija *et al*., 1994). Growers are frequently not aware of the importance of nematodes or the species present in their plantations (Hauser and Amougou, 2010). A lack of effective resistance genes and sterility both limit progress by conventional breeding, but sterility also prevents gene flow, strongly favouring a transgenic approach for nematode resistance (Lorenzen *et al*., 2010).

RNA interference (RNAi) has great potential for the development of transgenic crops with resistance to a range of pests and pathogens, as reviewed by Kaur *et al*. (2016). The RNAi effect was first described for *Caenorhabditis elegans* (Fire *et al*., 1998) and subsequently for plant‐parasitic nematodes (Urwin *et al*., 2002). Plants expressing dsRNA targeting nematode genes have been shown to suppress *Heterodera schachtii* (Patel *et al*., 2008, 2010; Sindhu *et al*., 2009), *H. glycines* (Klink *et al*., 2009; Li *et al*., 2010), *Meloidogyne* sp. (Huang *et al*., 2006; Ibrahim *et al*., 2011; Yadav *et al*., 2006) and *R. similis* (Li *et al*., 2015a,b). Additionally, *in vitro* treatment of *P. coffeae*,* P. thornei* and *P. zeae* with dsRNA targeting troponin C (*pat‐10*) or a calponin (*unc‐87*) resulted in aberrant movement and reduced multiplication on carrot discs (Joseph *et al*., 2012; Tan *et al*., 2013).

Optimization of dsRNA is required to both ensure that the intended target genes are silenced and to decrease the likelihood of off‐target effects. The exact level of conservation of sequence required to initiate RNAi has yet to be clearly defined. Exact matches between siRNAs generated from a dsRNA and target transcript over 19 nucleotides (nt) have been reported as sufficient for silencing (Naito *et al*., 2005), although off‐target effects in mammals can be triggered by a 6‐nt seed at the 5′ end of the siRNA (Kamola *et al*., 2015). A study that progressively reduced the homology between dsRNA and target sequence suggested that there is a gradual reduction in efficacy rather than a sharp loss of effect (Parrish *et al*., 2000). Another potential aspect of dsRNA molecules that has been shown to impact efficacy is molecule length. Molecules may have to be at least 50 bp long in insects, with no effect when a 25‐bp molecule is used (Yang and Han, 2013). The position of the region targeted by a dsRNA may also affect the efficacy of RNAi. Molecules designed to target 3′ regions of a transcript have proven more potent than targeting 5′ regions in nematodes (Lilley *et al*., 2012), although this is not necessarily true in insects (Yang and Han, 2013). To control across species, sequence conservation of the target region is also an important consideration. Experimental testing of any *in silico* prediction is necessary because the criteria for RNAi efficacy are not fully defined.

The RNAi approach potentially reduces regulatory hurdles as it involves no expression of novel protein or peptide thereby enhancing its inherent biosafety (Parrott *et al*., 2010), although for large‐scale production and commercialization, a range of biosafety assessments are still required (Auer and Frederick, 2009; Heinemann *et al*., 2013; Roberts *et al*., 2015). The level of food and feed safety of RNAi defences is likely to be high due to extensive degradation, biological barriers to uptake and low efficacy of exogenous nucleic acids ingested by mammals (Petrick *et al*., 2013). Environmental risks to nontarget organisms such as nematodes and insects that feed on the transgenic plant can be managed. This requires appropriate genetic information to avoid of dsRNA sequences likely to induce off‐target effects (Rual *et al*., 2007; Yamada and Morishita, 2005). Alternative approaches are required when sequence of putatively at‐risk species is not available from databases. One strategy involves assessing cross‐hybridization of dsRNA molecules to representative model species with well‐characterized transcriptomes (Naito *et al*., 2005). A second is exposure of sentinel, environmental indicator species that are highly RNAi susceptible to the dsRNA (Custodia *et al*., 2001; Winston *et al*., 2007).

There is a great need for a rational defence against a proscribed range of pathogens without adverse effects on nontarget organisms. In this study, the control of *R. similis* and *P. coffeae* were prioritized as the most damaging pests of banana (Gowen *et al*., 2005) and *Meloidogyne* introduced to test broader resistance conferred by the dsRNA molecules of interest that had been identified in the initial phase. The dsRNA molecules identified in this study offer a first step in realizing the potential for a broadly based resistance to economically important nematode pests in an important staple crop. The approach is readily extendable to other crops with a range of nematode pests such as rice. Additionally, transformation of crop plants, and particularly banana, involves a considerable investment in both time and resources. We address this constraint by describing a framework to identify and validate biosafe dsRNA molecules that have the potential to enhance the rate of development of future RNAi defences for many crop pests and pathogens.

## Results

### Target identification

Bioinformatic comparison of *C. elegans* genes with severe RNAi phenotypes and conservation to the economically important banana nematodes *R. similis*,* P. coffeae*,* H. multicinctus* and *Meloidogyne incognita* identified nine genes that shared >80% sequence identity between *C. elegans* and at least two of the banana nematodes. Sequences for *C. elegans*,* R. similis* and *M. incognita* were obtained from publically available databases. Amplification from cDNA was used to obtain sequences for *P. coffeae* and *H. multicinctus* using primers designed to conserved regions identified from database sequences (Table 1). Sequences with sufficient conservation for dsRNA design across all four target nematodes were identified for Actin‐4 (*act‐4*) and proteasomal alpha subunit 4 (*pas‐4*) genes (Table 2). Double‐stranded RNA (dsRNA) molecules were designed to target regions of these two genes that had one or more exact 19 nucleotide sequence matches. These regions corresponded to a 376‐bp section within the substrate‐binding domain region of ACT‐4 and a 292‐bp section of the PAS‐4 N‐terminal nucleophile (Ntn)‐hydrolase active site. The sequences used were derived from cDNA of *P. coffeae* for *pas‐4* and *H. multicinctus* for *act‐4* (Figure S1). A search for potential off‐target effects of the two dsRNA sequences was completed using a database (http://dsCheck.RNAi.jp/; Naito *et al*., 2005). It identified 19mers matching five *C. elegans* genes, eight *Drosophila* genes, five rat genes and one rice gene. All *C. elegans* and rat matches were eliminated by truncating the *act‐4* sequence to 112 bp (*act‐4_t*). A 19mer match to one rice gene and matches to two *Drosophila* genes were still present in the truncated *act‐4* sequence. There were no nontarget organism 19mer matches to the *pas‐4* dsRNA molecule.

**Table 1:** Potential target genes identified from database sequences

Gene	Wormbase ID	*C. elegans* expression	*C. elegans* RNAi phenotype	Rs	Mi	Pc	Hm
*ACT‐4*	WBGene 00000066	Body wall and vulval muscles	Embryonic lethal	RSC00784	MIC00350	✓	✓
*CCO‐1*	WBGene 00000371	Mitochondrial	Lethal	RSC00550	–	✓	–
*HSP‐60*	WBGene 00002025	Mitochondrial	Embryonic lethal	RSC02271	MIC03015	–	–
*PAS‐3*	WBGene 00003924	All major muscles	Embryonic lethal	RSC00234	MIC05526	✓	–
*PAS‐4*	WBGene 00003925	Pharynx and body wall muscle	Lethal	✓	MIC06652	✓	✓
*RPS‐23*	WBGene 00004492	Muscles and nervous system	Embryonic lethal	RSC00184	MIC03023	–	–
*UBQ‐1*	WBGene 00006727	Muscles, hypodermis and nervous system	Lethal	RSC02466	MIC06345	–	✓
*UBQ‐2*	WBGene 00006728	Muscles and nervous system	Embryonic lethal	RSC00037	MIC06345	✓	–
*UNC‐87*	WBGene 00006819	All major muscles	Lethal	RSC04225	MIC03833	–	–

RNAi phenotypes are the most severe given on Wormbase for *C. elegans*. The Nembase 4 gene reference is given for *R. similis* and *M. incognita* sequences obtained following a BLAST search with the *C. elegans* sequence. Sequences amplified from cDNA either had >80% sequence identity and 21‐nt regions with (✓) or without (–) 100% identity to other banana nematode sequences. The plant‐parasitic nematodes are as follows: *R. similis* (Rs), *M. incognita* (Mi), *Pratylenchus coffeae* (Pc) and *Helicotylenchus multicinctus* (Hm).

**Table 2:** Sequence identity of *act‐4* and *pas‐4* dsRNA molecules to target gene sequences of five nematodes with the species to which they were cloned indicated (*ex‐*)

	*act‐4* (ex *H. multicinctus*)		*pas‐4* (ex *P. coffeae*)
Nematode	376 bp	112 bp	307 bp
*Radopholus similis*	90%	88%	100%
*Helicotylenchus multicinctus*	100%	100%	100%
*Pratylenchus coffeae*	90%	79%	100%
*Meloidogyne incognita*	81%	87%	96%
*Caenorhabditis elegans*	86–83% (5)	81–85% (5)	68%

Value ranges of sequence similarities are for the number of orthologues, as indicated in parentheses. The dsRNA sequence similarities to targeted genes are subdivided into three categories: (i) at least one 21 perfect nucleotide sequence match (no shading), (ii) one or more 40 nucleotide sequences that share 38–39 nucleotides (lighter grey shading) and (iii) other, lower levels of similarity (darker grey shading).

### *In vitro* dsRNA treatment

**Figure 1:**
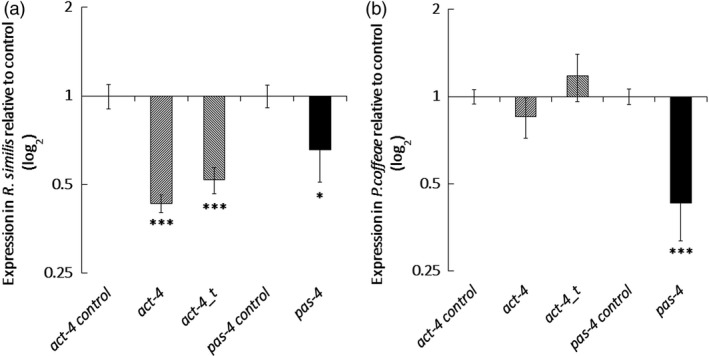
Quantitative RT‐PCR Measurement of Transcript Knock‐down. Reduction is for *act‐4* and *pas‐4* expression following soaking in 100 μg/mL dsRNA for 24 h for (a) *Radopholus similis* and (b) *Pratylenchus coffeae*. Values are means ± SEM for four technical replicates of three biological replicates with significant loss of expression relative to nontargeting *gfp* dsRNA treatment indicated. Analysis is based on univariate ANOVA of log values with Tukey *post hoc* comparison to *gfp*, ***P <* *0.001; *P <* *0.05.

**Figure 2:**
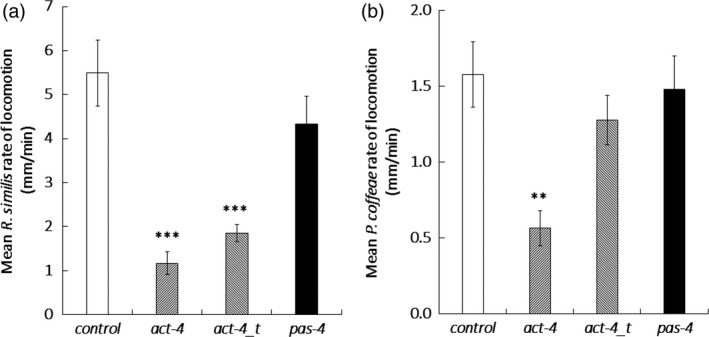
Effect of dsRNA treatment on nematode locomotion. Speed of locomotion (mm/min) was recorded for (a) *Radopholus similis* and (b) *Pratylenchus coffeae* on an agar surface after soaking in 100 μg/mL dsRNA targeting *act‐4* or *pas‐4*. Significance is given from *post hoc* comparison of each RNAi effect relative to the *gfp* dsRNA control. Analysis is based on univariate ANOVA of log values (*n* > 15) with Tukey *post hoc* comparison to *gfp*, ***P <* *0.001; **P <* *0.01.

Quantitative RT‐PCR established that dosing with dsRNA for 24 h resulted in a significant 1.5‐fold reduction of *pas‐4* transcript abundance (*P *<* *0.05, one‐way ANOVA with Tukey *Post hoc*) for *R. similis* and 2.3‐fold (*P *<* *0.001) for *P. coffeae* relative to the *gfp* dsRNA control treatment. Both the *act‐4* long dsRNA and truncated dsRNA molecules significantly suppressed *act‐4* transcript in *R. similis* by 2.3‐ and 1.9‐fold, respectively (*P *<* *0.001), but neither had a significant effect on *P. coffeae* transcript levels (Figure 1). The *act‐4* targeting molecules decreased the rate of locomotion of *R. similis* by 79 ± 9% for the 376‐bp molecule and 66 ± 4% for the 112‐bp molecule (*P *<* *0.001) relative to control nematodes (Figures 2a and Video S1). The longer *act‐4* molecule also reduced the locomotion of *P. coffeae* by 64 ± 7% (*P *<* *0.001), but the truncated version did not (Figures 2b and Video S1). Targeting *pas‐4* had no significant effect on the rate of locomotion of either nematode (Video S1).

### Multiplication of *R. similis* and *P. coffeae* on carrot discs

**Figure 3:**
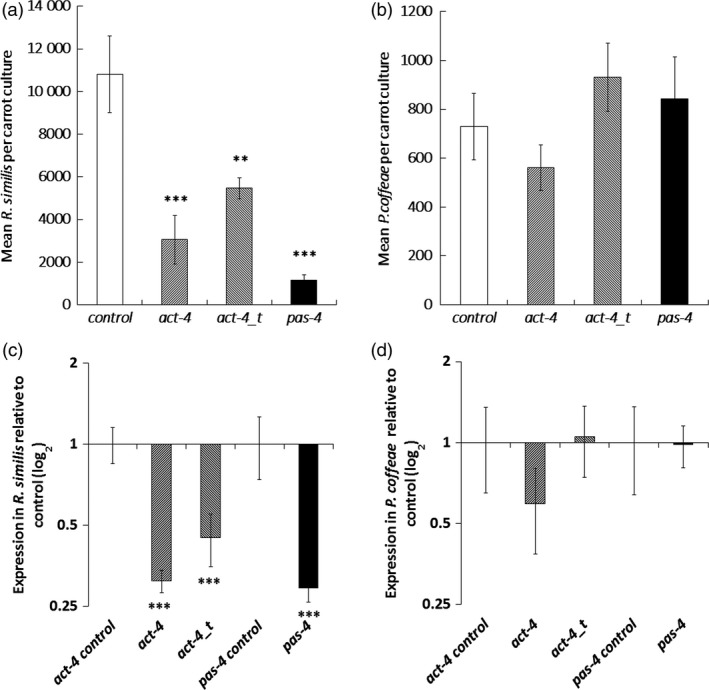
Effect of dsRNA treatment of nematode cultured on carrot discs. The effect of dosing nematodes weekly with dsRNA molecules on (a,b) mean population density and (c,d) target transcript level was assessed after 8‐week culture on carrot discs for (a,c) *Radopholus similis* and (b,d) *Pratylenchus coffeae*. Dosing was with dsRNA molecules in M9 buffer targeting sequences of *act‐4* (*act‐4* and *act‐4_t*) or *pas‐4*. Quantitative RT‐PCR values are means ± SEM for four technical replicates of three biological replicates with significant loss of expression relative to nontargeting *gfp* dsRNA treatment indicated. Analysis is based on univariate ANOVA of log values (*n* = 10) with Tukey *post hoc* comparison to *gfp*, ***P <* *0.001; **P <* *0.01.

Long‐term effects of the three dsRNA molecules were studied by weekly dosing during culture on carrot discs over 8 weeks. The long and truncated *act‐4* dsRNA molecules suppressed *R. similis* population increase by 72 ± 11% (*P *<* *0.001) and 49 ± 5% (*P *<* *0.01), respectively, and the *pas‐4* targeting dsRNA by 89 ± 2% (*P *<* *0.001) relative to controls (Figure 3a). *R. similis* nematodes recovered from the carrot discs at 8 weeks also had significant reduction in targeted transcript (Figure 3c) No significant reduction occurred in multiplication or transcript levels of *P. coffeae* individuals with any of the three dsRNA treatments (Figure 3b,c).

### Transgenic hairpin carrot hairy roots

The *in planta* suppression of *R. similis*,* P. coffeae* and *M. incognita* when targeting *act‐4* or *pas‐4* was determined using transgenic carrot hairy roots expressing hairpin constructs for dsRNA production. An initial challenge with *R. similis* was carried out with three replicates of 10 RT‐PCR‐positive independent transformation events per construct, including a dsRNA *gfp* construct as a null target control. This screen identified four lines, *act‐4_t* lines 5 and 6, *pas‐4* lines 3 and 4, which were most effective at suppressing nematode multiplication. Nine *act‐4_t* lines and four *pas‐4* lines provided >50% resistance. Additionally, two control *gfp* lines were chosen at random for subsequent challenges.

**Figure 4:**
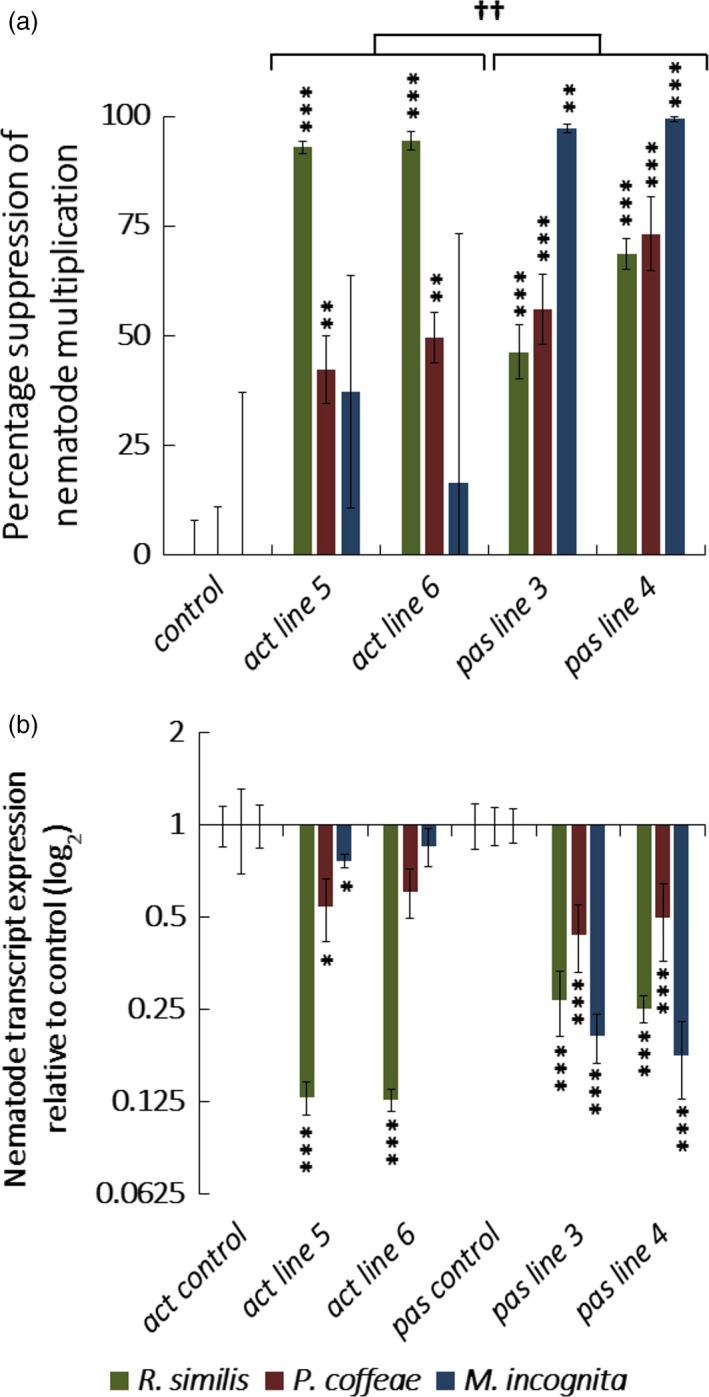
Transgenic Carrot Hairy Root Challenges. (a) Percentage suppression of multiplication obtained and (b) fold transcript knock‐down for nematodes recovered from hairy root lines expressing dsRNA targeting *act‐4*,* pas‐4* or *gfp* after 10 weeks of culture when challenged with *Radopholus similis*,* Pratylenchus coffeae* or *Meloidogyne incognita*. Analysis is based on one‐way ANOVA of log values (*n* = 15) with Tukey *post hoc* comparison of individual lines to *gfp* control (***P <* *0.001; **P <* *0.01; *P <* *0.05) and *a priori* ANOVA both *act‐4*_t lines to both *pas‐4* lines (^††^ *P *<* *0.01).

Nematode multiplication (Figure 4a) was significantly reduced (*P *<* *0.001) for *R. similis* by 93 ± 1% and 94 ± 2% on *act‐4_t* lines 5 and 6 and 46 ± 6% and 69 ± 3% on *pas‐4* lines 3 and 4. Transcript levels of the targeted genes (Figure 4b) in *R. similis* were also significantly suppressed (*P *<* *0.001) 7.7‐fold and 7.9‐fold for *act‐4_t* lines 5 and 6 and 3.7‐fold and fourfold for *pas‐4* lines 3 and 4. Multiplication of *P. coffeae* was also significantly reduced (*P *<* *0.01) on all four lines, 42 ± 8% and 50 ± 6% on *act‐4_t* lines and 56 ± 8% and 73 ± 8% on *pas‐4* lines. Nematode transcript abundance for *act‐4_t* line 5 was 1.8‐fold reduced (*P *<* *0.01) and 2.3‐fold and twofold for *pas‐4* lines 3 and 4 (*P *<* *0.001). There was no significant reduction in the number of *M. incognita* J2s recovered from either *act‐4_t* line while a small but significant 1.3‐fold reduction in transcript abundance was detected for line 5 (*P *<* *0.01). In contrast, the two *pas‐4* lines reduced the multiplication of this nematode by 97 ± 1% and 99 ± 1% (*P *<* *0.001) and transcript abundance by 4.9‐ and 5.6‐fold (*P *<* *0.001). There was a significant difference (*P *<* *0.05) between the relative efficacy of the two constructs against the different nematode species. *R. similis* was more disrupted by *act‐4_t* whereas *pas‐4* was more effective against *P. coffeae* and *M. incognita*.

### Effect on nontarget nematodes

**Figure 5:**
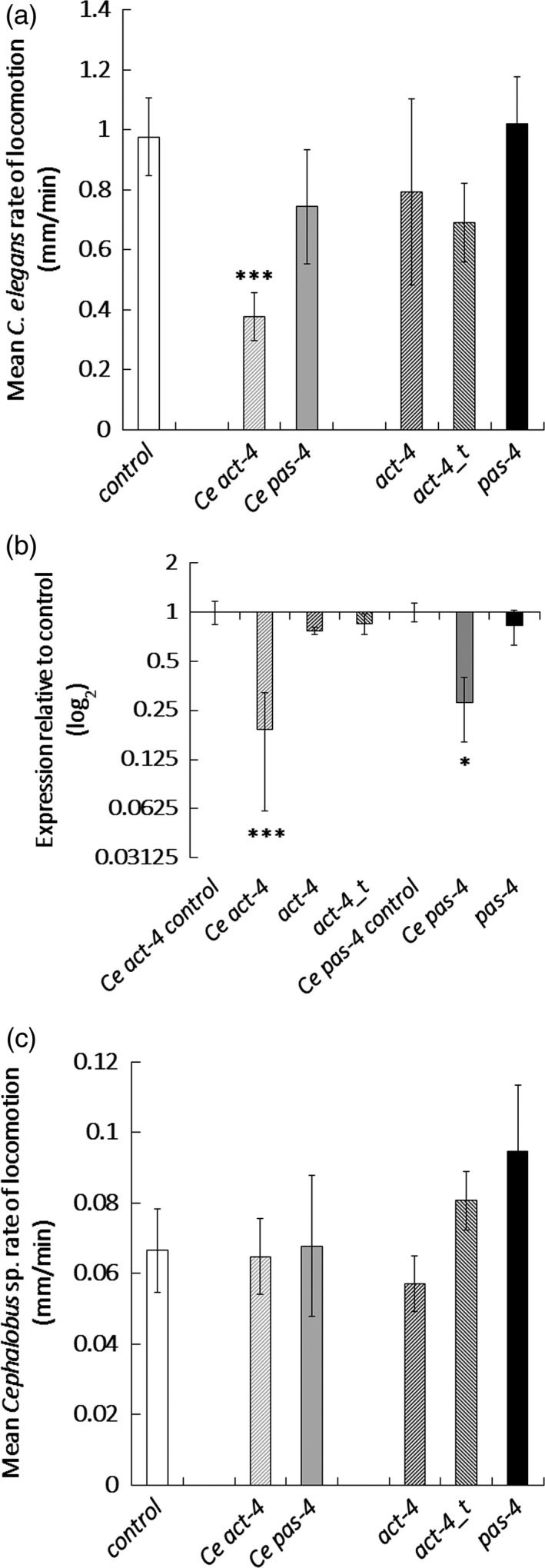
Effect of dsRNA treatment on nontarget nematodes. (a) Mean rate of locomotion and (b) transcript knock‐down was recorded for active *Caenorhabditis elegans* nematodes soaked for 24 h in 100 μg/mL dsRNA targeting sequences specific to *C. elegans* (Ce) or plant‐parasitic nematodes and (c) mean rate of locomotion obtained for *Cephalobus* sp. soaked for 24 h in 100 μg/mL dsRNA targeting sequences specific to *C. elegans* (Ce) or plant‐parasitic nematodes. Analysis is based on univariate ANOVA of log values (*n* > 15) with Tukey post hoc comparison to *gfp*, ***P <* *0.001; *P <* *0.05.

A faunal analysis identified *Caenorhabditis* and *Cephalobus* species in soil samples from a transgenic banana field trial site at Kawanda, Uganda. Both species readily took up fluorescein isothiocyanate (FITC) *in vitro* (data not shown) and were selected as suitable sentinel species for an off‐target study. *In vitro* treatment with the *act‐4* dsRNA molecules designed to target plant‐parasitic nematodes did not reduce locomotion of *C. elegans* in contrast to its counterpart directed at the endogenous *act‐4* gene (Figure 5a). Both this dsRNA and one targeted at *pas‐4* of *C. elegans* significantly reduced expression (*P *<* *0.001; Figure 5b) in contrast to when this nematode was soaked in the banana nematode dsRNA variants. None of the dsRNA molecules targeted at either plant‐parasitic nematodes or *C. elegans* reduced the rate of locomotion of *Cephalobus* (Figure 5c).

## Discussion

This work identifies dsRNA sequences capable of cross‐genera control of economically important crop pests. Transcripts that have lethal phenotypes when knocked out in *C. elegans* and conservation across plant‐parasitic nematodes were targeted with dsRNA *in vitro* and *in vivo*. Control was achieved by targeting *pas‐4*, a proteasome alpha‐type seven subunit of the core 20S proteasome subcomplex involved in protein turnover (Papaevgeniou and Chondrogianni, 2014), and *act‐4*, an actin gene fundamental to muscle function. Both genes are predominantly expressed in *C. elegans* body wall muscles.

Targeting *pas‐4* suppressed parasitism and transcript on transgenic hairy roots for *R. similis*,* P. coffeae* and *M. incognita*. The effectiveness of the *pas‐4* dsRNA molecule when expressed *in planta* against all three banana nematodes may be due to its high conservation between all plant‐parasitic nematodes. The lack of a significant effect *in vitro* on *P. coffeae* may reflect that weekly dosing with dsRNA on carrot discs is insufficient to maintain the RNAi effect achieved relative to continual exposure *in vivo*. By contrast, targeting *pas‐4* suppressed multiplication of *in vitro*‐dosed *R. similis* on carrot discs by 89 ± 2%. The suppression of transcript levels in *R. similis* recovered from carrot discs and the lack of suppression in *P. coffeae* suggest that the higher activity of *R. similis* and hence increased rate of feeding than *P. coffeae* (Stoffelen *et al*., 1999) results in uptake of sufficient dsRNA to maintain an RNAi effect while cultured on carrot discs. Despite high expression of the *pas‐*4 in body wall muscles and a large knock‐down following *in vitro* treatment with dsRNA, there was no effect on nematode locomotion. *C. elegans*‐fed bacteria expressing *pas‐4* dsRNA had uncoordinated movement in a single experiment but not in all experiments of the study (Simmer *et al*., 2003). There was also no effect on locomotion in our experiments with plant‐parasitic nematodes or *C. elegans* treated with dsRNA in solution. This may indicate an effect on the locomotory or other musculature, such as those in the pharynx required for feeding, that only becomes evident following consistent and long‐term exposure when nematodes are cultured on transgenic roots.

The 376‐bp *act‐4* dsRNA molecule had four regions with >19 nt sequence identity with the *R. similis act‐4* transcript, including one in the truncated dsRNA region, and suppressed its locomotion and multiplication after oral dosing, during culture on carrot discs and when a parasite on transgenic hairy roots. The results for *P. coffeae* were somewhat different. No effect was detected with the truncated *act‐4* dsRNA molecule. The locomotion of *P. coffeae* orally treated with the 376‐bp *act‐4* dsRNA molecule was suppressed, but the small reduction in its *act‐4* transcript was not statistically significant. Transgenic *act‐4* line 6 also showed a phenotypic effect of significantly reduced multiplication without the accompanying small fall in message abundance reaching statistical significance in contrast to *act‐4* line 5. Possibly, even a small reduction in transcript abundance has phenotypic effects on *P. coffeae*. The truncated 122‐bp molecule reduced *P. coffeae* multiplication on transgenic roots despite lacking comparatively low sequence identity to the target sequence. Possibly, the strength of base pairing of nucleotide seed match in positions 2 to 7–8 nucleotides of the antisense strand of the siRNA (Birmingham *et al*., 2007) is a stronger predictor of a potential effect than overall sequence identity between dsRNA molecule and transcript target sequence. This matching did occur between the *P. coffeae* sequence and potential *act‐4_t* seed regions. The efficacy of the truncated version of the *act‐4* dsRNA with a comparatively low sequence similarity to the actin genes and the lack of an effect in *C. elegans* despite the presence of >19nt exact sequence matches are both of interest. They indicate the importance of experimental confirmation of *in silico* analyses given a higher sequence similarity of at least 88% has been suggested as required for an RNAi effect (Parrish *et al*., 2000).

The strategy of identifying single dsRNA molecules that target conserved sequences across nematode genera probably increased the chances of selecting genes with important biological roles and hence a significant RNAi knock‐down phenotype. Identification of targets for *R. similis* and *M. incognita* were greatly aided by genetic information for these nematodes (Abad *et al*., 2008; Bird *et al*., 2015; Jacob *et al*., 2009; Opperman *et al*., 2008). The recently published genome of *P. coffeae* (Burke *et al*., 2015) should also prove useful for future work. A constraint on target identification in this work was the lack of genetic information for *H. multicinctus* and, at the time of selecting targets, *P. coffeae*. Identification of homologous sequences for these species relied on amplification with primers designed to conserved regions in *C. elegans*,* R. similis* and *M. incognita* of the genes of interest. Additionally, a constraint on *in vitro* screening using carrot discs is the inability to culture *H. multicinctus*, as carrot is not a host for that nematode, or *M. incognita*, which cannot be cultured on carrot discs. Another issue, discussed above, is an inability to ensure consistent exposure to the dsRNA. As such, this screen is likely to underestimate the effectiveness of an RNAi effect as compared to the transgenic root screen.

The availability of complete genetic sequences not only aids the identification of RNAi targets but also allows the assessment of off‐target potential. The transformed dsRNA sequences had no 21‐nt matches for carrot in GenBank or the published banana genomes (D'Hont *et al*., 2012; Davey *et al*., 2013). The dsCheck software was used to reduce off‐target potential of the dsRNAs designed for this work. It provides an accelerated off‐target search algorithm for siRNAs to identify exact and near nucleotide matches to the five representative genomes of *Oryza sativa* (Rice), *Arabidopsis thaliana*,* Rattus norvegicus* (Rat), *Drosophila melanogaster* and *C. elegans* (Naito *et al*., 2005). There are also no exact 21‐nt matches of the transformed dsRNA sequences to the ESTs available in GenBank. *In silico* sequence analysis provides a useful means of selecting molecules for further study and supports rational design to reduce likely risk. However, the potential hazard of transitive silencing along the target sequences and production of secondary siRNAs that could possible cause off‐target effects must be evaluated. It requires transgenic lines of the intended crop, in this case banana, because the risk of off‐target effects is heavily dependent on the context of RNAi molecules. Consequently, the assessment off‐target effects of RNAi molecules is a future research need as hairy roots of carrot cannot indicate the likelihood of such effects in other plants.

There is an inherent advantage to any approach that can replace the low specificity and high toxicity of current nematode control chemicals. There is, however, a possibility of an RNAi effect on a wide range of nontarget organisms due to the high conservation of these two target transcripts that requires consideration in the design of dsRNA molecules. Previous studies have concentrated on parasitism genes to enhance the safety of the approach either by design (Huang *et al*., 2006) or through a process of excluding genes with homology in nontarget organisms (Danchin *et al*., 2013). The disadvantage of this approach is that target genes will likely be too specific for a broad control of crop pests. A more fine‐grained approach provides a larger pool of potential targets from which to choose. It bases selection on the homology of a targeted gene region, as opposed to the homology of the entire gene. This increases the potential for the control of a range of nematodes of several genera with different modes of parasitism, as demonstrated in this work, and ensures a low homology to transcripts in nontarget organisms, including humans. A lack of food hazard is also suggested by a wide range of evidence that dsRNA is not a hazard in the human diet (Petrick *et al*., 2013). We consider this provides a *prima facie* case for the food safety of the antinematode RNAi defences. Several steps in risk assessment are essential for both food and environmental safety once transgenic events of interest are identified. A key need is banana plants and fruit for the recommended context‐specific safety evaluation *in planta* of a transgenic event (Arpaia *et al*., 2017). That approach is required by regulatory authorities for potential nontarget effects for each transformation event generated (e.g. EFSA, 2010). One appropriate approach is the comparative safety assessment paradigm as employed for already commercialized transgenic crops (Parrott *et al*., 2010; Petrick *et al*., 2013). Biosafety evaluations are planned in both glasshouses and the field for crop plant growth, agronomic characteristics and environmental impact on nontarget organisms selected by a range of criteria (Ahmad *et al*., 2016), as well as food safety assessments on fruit.

Environmental monitoring should be used to complement the preventative design strategy, particularly where genetic information for at‐risk organisms is lacking. The emphasis of environmental risk assessments often focuses on beneficial organisms (Romeis *et al*., 2011), particularly those related to the target pest. Therefore, beneficial nematodes in the soil around the crop need to be considered when plant‐parasitic species are targeted for RNAi. *Caenorhabditis* and *Cephalobus* were selected for this work as both readily took up fluorescent molecules *in vitro*, unlike other free‐living nematodes tested. Both genera occurred in soil from a banana field in Uganda. Soaking *C. elegans* in dsRNA molecules designed to target its own *act‐4* and *pas‐4* genes mirrored the response of the parasitic nematode with suppression of transcript of both genes and a reduction in locomotion of *act‐4* dsRNA‐treated nematodes. Transcript abundance and locomotion in *C. elegans* were not affected by the sequences targeted at the banana nematodes which have 85% identity for *act‐4_t* and 68% identity for *pas‐4* to the *C. elegans* sequences. They may have provided too few exo‐siRNAs to trigger sufficient production by *C. elegans* of the secondary siRNAs that are required to amplify the signal (Billi *et al*., 2014). Locomotion of *Cephalobus*, a slow‐moving nematode, was not adversely affected by any of the dsRNAs. Previous work suggests many genera of free‐living soil nematodes are less readily affected by dsRNA than *C. elegans* (Wheeler *et al*., 2012). Sentinel species are a well‐established approach to indicate potential hazards (van der Schalie *et al*., 1999). Our results suggest *C. elegans* is appropriately susceptible for this use in monitoring RNAi‐mediated hazards to nontarget nematodes.

These results establish a framework for selecting gene targets for RNAi‐based control of crop pests that are from several distinct genera. This work concentrates on nematode pests of banana, but the approach is equally applicable to any group of pests and pathogens for which common and effective target sequences can be identified. Genetic resources and screens for efficacy are available for the approach to be extended to insects (Joga *et al*., 2016), viruses (Galvez *et al*., 2014) and fungi (Salame *et al*., 2011).

## Experimental procedures

### Identifying sequences of interest

Genes of interest were identified by searches of the GenBank database (http://www.ncbi.nlm.nih.gov; Benson *et al*., 2013), the NEMBASE4 EST database (http://www.nematodes.org/nembase4/; Elsworth *et al*., 2011), the nematode.net EST database (http://www.nematode.net/; Martin *et al*., 2015) and Wormbase (http://www.wormbase.org/ release WS227; Howe *et al*., 2016). The search was for gene EST sequences present in more than one of *R. similis*,* Pratylenchus* spp., *H. multicinctus* and *Meloidogyne* spp. and with a severe RNAi phenotype in *Caenorhabditis elegans*. Alignments using Clustal Omega version 1.2.3 (http://www.ebi.ac.uk/Tools/msa/clustalo/; Sievers *et al*., 2011) of homologous gene sequences were used to identify regions for primer design with high identity to ensure amplification from all targeted species. These primers were designed using Primer3Plus (http://primer3plus.com/cgi-bin/dev/primer3plus.cgi; Untergasser *et al*., 2012) and used to amplify gene sequences by standard PCR from nematodes where a database sequence was not available. Sequences were cloned into pCR‐Blunt II‐TOPO (Invitrogen, Carlsbad, CA) and sequenced from the M13 forward and reverse primer sites on that plasmid.

### Generating dsRNA expressing constructs

DNA fragments of the target genes were amplified by PCR using specific primers with restriction site linkers (Table S1) to generate expression vectors. A modified GFP sequence (Haseloff *et al*., 1997) was used in to provide the nontarget control treatment. For *in vitro* experiments, the DNA fragments were subcloned into the TA‐cloning vector pCR‐Blunt II‐TOPO (Invitrogen) and then cloned into the *Nhe*I site of the vector L4440 (pPD129.36; Timmons and Fire, 1998). The resultant constructs were verified by DNA sequencing.

Hairpin vectors were generated with the same DNA fragments. They were cloned in a sense and antisense orientation either side of the spacer (loop) intron in the pHANNIBAL vector (Wesley *et al*., 2001). The *act‐4_t* fragment was cloned 5′–3′ into the *Xho*I and *Kpn*I sites and 3′–5′ into the *Xba*I and *Hin*dIII sites of the vector. The *pas‐4* fragment was cloned 5′–3′ into the *Eco*RI and *Kpn*I sites and 3′–5′ into the *Xba*I and *Hin*dIII sites of the vector. The hairpin cassette containing, in 5′–3′ order, the CaMV35S promoter, the sense orientation target fragment, the spacer intron, the antisense orientation target fragment and an *OCS* 3′ region was then ligated into the *Not*I site of the pART27 binary vector (Gleave, 1992). Vector sequences were checked by sequencing then transformed into *Agrobacterium rhizogenes* strain R1000 (Chilton *et al*., 1982) by the freeze–thaw method.

### Nematode culture

*Radopholus similis* and *P. coffeae* were maintained on carrot disc cultures and were collected by washing the carrot discs in Petri dishes with 15 mL tap water overnight. The water was transferred to 15 mL tubes and the nematodes allowed to settle for 1 h before transfer to a 1.5‐mL microcentrifuge tube using a glass pipette. *H. multicinctus* and *M. incognita* were maintained on banana plant roots and collected from chopped banana roots using a misting chamber (Southey, 1986) into 50‐mL tubes that were changed every 24 h for 3 days. Nematodes collected at the bottom of the tube were transferred to 1.5‐mL microcentrifuge tubes with a glass pipette.

The bacterial‐feeding nematode *Cephalobus* sp. wild isolate (strain DWF1301) was provided by CGC, which is funded by NIH Office of Research Infrastructure Programs (P40 OD010440). *Cephalobus* sp. and *C. elegans* nematodes were maintained on lawns of *Escherichia coli* strain OP50 on Nematode Growth Medium (NGM) agar plates (Dusenbery *et al*., 1975) and collected by washing plates with 2 mL sterile tap water for 15 min and collecting nematodes into 1.5‐mL microcentrifuge tubes.

Prior to all experiments, nematodes were surface‐sterilized with 1 mL of sterilization mix (0.1% kanamycin, 0.1% penicillin G, 0.1% streptomycin sulphate, 50 μg/mL amphotericin B, 0.1% cetyltrimethylammonium bromide [CTAB]) for 30 min followed by five washes with 1 mL sterile tap water.

### *In vitro* uptake of dsRNA

Synthesis of complementary single‐stranded RNAs (ssRNAs) was from the T7 promoters located either side of the multiple cloning site (MCS) in each nematode targeting L4440 construct following independent digestion with *Kpn*I and *Sac*I. Synthesis of ssRNA, annealing to dsRNA and dsRNA purification were by Ambion Megascript T7 RNAi kit (Invitrogen) according to the instructions provided. Three replicates of 200 nematodes were soaked in 100 μg/mL dsRNA in M9 buffer with 100 mm Octopamine at 25 °C in the dark for 24 h in 1.5‐mL tubes. This neurotransmitter has been used before as described for both *Pratylenchus* and *Meloidogyne* without adverse effects (Rosso *et al*., 2005; Tan *et al*., 2013).

### Locomotion assay

Nematodes treated with dsRNA or collected from carrot hairy roots were aliquoted onto water agar plates (1% agar, 0.25% HEPES, 0.25% Tween‐20, pH 7.2). Video capture of locomotion of >15 active nematodes per treatment over 30 s was recorded at four frames per second and 1.25× magnification using a Q Imaging Micropublisher 3.3 RTV camera attached to a Leica M165C stereomicroscope. Track lengths of the imaged nematodes were measured over at least 30 s on each occasion using the wrMTrck plugin (Nussbaum‐Krammer *et al*., 2015) for Image J (Schneider *et al*., 2012) version 1.46r and exported to an Excel worksheet for subsequent analysis.

### Carrot disc culture

Sterile nematodes treated for 16 hr with 100 μg/mL dsRNA were transferred to sterile carrot discs on 1% agar, 10 replicates of 250 nematodes per culture, and incubated at 25 °C in the dark for 56 days. Every 7 days, 100 μg/mL dsRNA in 1 ml M9 buffer was applied to each carrot disc culture. Nematodes were collected by washing with tap water, counted and half were used for the locomotion assay and half flash‐frozen for quantitative RT‐PCR analysis.

### Hairy root cultures

Hairy root cultures were established on Petri dishes of 1% agar modified White's medium (MW; Becard and Fortin, 1988) with 50 μg/mL kanamycin selection from transformed carrot discs as described by Cardarelli *et al*. (1987). Expression of hairpin constructs was confirmed by RT‐PCR using primers designed to amplify the dsRNA molecules with amplification of the carrot actin‐7‐like gene as an internal control (Table S2). Cultures with root extension rates of 10 ± 2 mm/week were selected to provide 10 transformation events per construct (*gfp*,* act‐4_t* and *pas‐4*). An initial screen to identify promising lines used three replicate hairy root cultures per transformation event, subsequent screens on the best lines were with 15 replicate cultures per transformation event. For nematode challenge, approximately 200 nematodes were inoculated onto the agar next to several growing root tips and incubated on the roots at 25 °C in the dark for 12 weeks. Mixed stage *R. simlis* and *P. coffeae* and *M. incognita* J2s were washed from the hairy root cultures using sterile tap water, counted and flash‐frozen for quantitative RT‐PCR analysis.

### Quantitative RT‐PCR

RNA was extracted from collected nematode samples by SV Total RNA Isolation System (Promega, Fitchburg, WI), which included an on‐column DNase treatment. Synthesis of cDNA was from 1 μg total RNA with 200 units Superscript II Reverse Transcriptase, and Oligo (dT) primers (Invitrogen). A one in 10 dilution of cDNA (100 ng/reaction) was used as template for qPCR analysis with gene‐specific primers with efficiencies >90% and <110% (Table S2) added to Agilent Brilliant II SYBR Green Master Mix (Agilent, Santa Clara, CA, US). Cycling conditions were 5 min at 95 °C followed by 40 cycles of 95 °C for 10 s and 55 °C for 30 s. A dissociation curve analysis was carried out at the end of each qPCR experiment to monitor reactions for nonspecific amplification. Quantification was the mean of four technical replicates for each of three biological replicates per experiment and was normalized to cell division control protein 42 (*cdc‐42*) for *R. similis*, heat‐shock protein 90 (*hsp‐90*) for *P. coffeae*, 18S ribosomal RNA sequences for *M. incognita* and *C. elegans*. Experiments were carried out using an Mx3005p qPCR Cycler (Agilent) and analysed in MxPro qPCR software (Agilent).

## Conflict of interest

The authors declare they have no conflict of interest.

## Supporting information


**Figure S1** Alignments of (a) *act‐4* and (b) *pas‐4* dsRNA molecules and their targets in the plant‐parasitic nematodes *Radopholus similis*,* Pratylenchus coffeae*,* Helicotylenchus multicinctus* and *Meloidogyne incognita* and the nontarget, *Caenorhabditis elegans*.
**Table S1** Primers used for dsRNA construct cloning. Linker restriction sites used for cloning are indicated in bold italics.
**Table S2** Primers used for transcript screening and quantification. Genes used as internal controls for normalising quantification indicated in bold.

**Video S1** Movement of *Radopholus similis* and *Pratylenchus coffeae* across 1% agar plates following treatment with two dsRNA molecules targeting act‐4, a dsRNA molecule targeting pas‐4 or a non‐targeting *gfp* sequence. Each nematode is representative of the mean distance moved for that species and treatment (N>15). Scale bar = 500μm.
