# Supplementary material for: Rational design of biosafe crop resistance to a range of nematodes using RNA interference
Source: Plant Biotechnol J. 2017 Aug 22;16(2):520–9. doi: 10.1111/pbi.12792 (PMC5787825; doi:10.1111/pbi.12792)

**Supplementary Information**

**Supplementary Table 1.** Primers used for dsRNA construct cloning. Linker restriction sites used for cloning are indicated in bold italics.

| **Gene** | **Primer Sequences** | **Product Size (nt)** |
| --- | --- | --- |
| *act-4* (L4440) | F: GTATCG***GCTAGC***AATTCAGGCAGAGGACGGTG  R: GTATCG***GCTAGC***GATCCACTAGTAACGGCCG | 400 |
| *act-4_t* (L4440) | F: GTATCG***GCTAGC***GTGATCACCGTCGGCAAC  R: GTATCG***GCTAGC***TCATGATGGAGTTGTAGGTGGA | 136 |
| *act-4_t* sense (pHANNIBAL) | F: GTATCG***GAATTC***TCATGATGGAGTTGTAGGTGGA  R: GTATC***GGTACC***GTGATCACCGTCGGCAAC | 135 |
| *act-4_t* antisense (pHANNIBAL) | F: GTATCG***TCTAGA***TCATGATGGAGTTGTAGGTGGA  R: GTATCG***AAGCTT***GTGATCACCGTCGGCAAC | 136 |
| *pas-4* (L4440) | F: ACTCCA***GCTAGC***GAGCACATCTCCCGCACTAT  R: ACTCCA***GCTAGC***CCATATTACGCGCTCCAGTT | 318 |
| *pas-4* sense (pHANNIBAL) | F: GCATTA***GGTACC***GCACTATTGCGGACTTGAAACA  R: GCATTA***CTCGAG***CCATATTACGCGCTCCAGTT | 316 |
| *pas-4* antisense (pHANNIBAL) | F: GCATTA***TCTAGA***GCACTATTGCGGACTTGAAACA  R: GCATTA***ATCGAT***CCATATTACGCGCTCCAGTT | 316 |
| *gfp* (L4440) | F: GCATTA***GCTAGC***GCACTATTGCGGACTTGAAACA  R: GCATTA***GCTAGC***CCATATTACGCGCTCCAGTT | 503 |
| *gfp* sense (pHANNIBAL) | F: ATGCTA***CTCGAG***TTTCTCTTATGGTGTTCAATGCTT  R: ATGCTA***GGTACC***CAGCTGTTACAAACTCAAGAAGGA | 503 |
| *gfp* antisense (pHANNIBAL) | F: ATGCTA***AAGCTT***TTTCTCTTATGGTGTTCAATGCTT  R: ATGCTA***TCTAGA***CAGCTGTTACAAACTCAAGAAGGA | 503 |
| C. elegans *act-4* (L4440) | F: AGTTGC***GCTAGC***AGAAGCTCTGCTATGTCGCC  R: AGTTGC***GCTAGC***CCTGGGTACATGGTGGTTCC | 283 |
| C. elegans *pas-4* (L4440) | F: AGTTGC***GCTAGC***CGAGTGCCAGTCGTACAAGT  R: AGTTGC***GCTAGC***ACCTGAGCCAGCGACTTTAC | 327 |

**Supplementary Table 2.** Primers used for transcript screening and quantification. Genes used as internal controls for normalising quantification indicated in bold.

| **Species** | **Gene** | **Primer Sequences** | **Product Size (nt)** |
| --- | --- | --- | --- |
| *Radopholus similis* | ***cdc-42*** | F: ACAAGTCGATTTGCGCGAAG  R: TGACGGCTTTCAGCTCTTTG | 113 |
|  | *act-4* | F: GTTACTCGTTCACCACCA  R: CAACTCGTGGCTCTTCTC | 137 |
|  | *ubq-1* | F: CGGAAAGCAGCTCGAGGAC  R: CGTCATATTCCTCGTTGCGTTC | 128 |
|  | *pas-4* | F: TTACTTGCTGGCTCAATCGG  R: TTGCACAACTGGGACAAGAG | 135 |
| *Pratylenchus coffeae* | ***hsp-90*** | F: ACGAGGAATACGCCGAATTC  R: AAGTGCTTCACCGCCAAATG | 70 |
|  | *act-4* | F: TCCACCTTCCAGCAAATGTG  R: ATTTGCGGTGCACAATGGAC | 73 |
|  | *ubq-1* | F: TTACTTGCTGGCTCAATCGG  R: TTGCACAACTGGGACAAGAG | 135 |
|  | *pas-4* | F: TTACTTGCTGGCTCAATCGG  R: TTGCACAACTGGGACAAGAG | 135 |
| *Meloidogyne incognita* | ***18S*** | F: ACTTGACGGGAGCATAATCG  R: CGGCCTCAAAGAGAACAGTC | 184 |
|  | *act-4* | F: CTTCCAGCCATCTTTCTTGG  R: CATTGTTGATGGTGCCAAAG | 193 |
|  | *ubq-1* | F: GTGGGATGCAGATCTTTGTG  R: CAATGGTATCAGACGCTTCC | 75 |
|  | *pas-4* | F: TTTGAGTGCAGATGCTCGTG  R: AGATGTGCTCAAGTGTGACG | 98 |
| *Caenorhabditis elegans* | ***18S*** | F: TTGCCCTTAAACGAGGAATG  R: GTACAAAGGGCAGGGACGTA | 73 |
|  | *act-4* | F: ACCCAATTGAGCACGGAATC  R: TTGGCCTTTGGATTGAGTGG | 139 |
|  | *ubq-1* | F: ATGGACGCACTCTATCCG  R: TAAATGTCTCCTCCGCGAAG | 82 |
|  | *pas-4* | F: TCGCGGAAAGGATTGCATTG  R: TGCGGACAATCCAGCAAAAG | 130 |
| Transgenic Carrot RTPCR | ***Dc act*** | F: CCCAATTGAACACGGCATTG  R: TGCTTTTGGGTTCAGTGGTG | 87 |
|  | *gfp* | F: ACTTCTTCAAGAGCGCCATG  R: TGACTTCAGCACGTGTCTTG | 102 |
|  | *act-4* | F: TTGTAGGTGGACTCGTGGATG  R: CAACGAGCGATTCCGTTGC | 93 |
|  | *pas-4* | F: TCTCATTCCGGCCGATTGAG  R: TTGTTGGCGGTTTTGATCCC | 133 |
|  |  |  |  |
|  |  |  |  |
|  |  |  |  |
|  |  |  |  |

**Supplementary Figure 1.**

Alignments of dsRNA molecules and their targets in the plant parasitic nematodes *R. similis*, *P. coffeae*, *H. multicinctus* and *M. incognita* and the non-target *C. elegans*. Grey regions indicate exact matches of ≥19nt between the target sequence and the dsRNA molecule. Black regions in the dsRNA sequence indicate truncated dsRNA sequence targeting *act-4*.


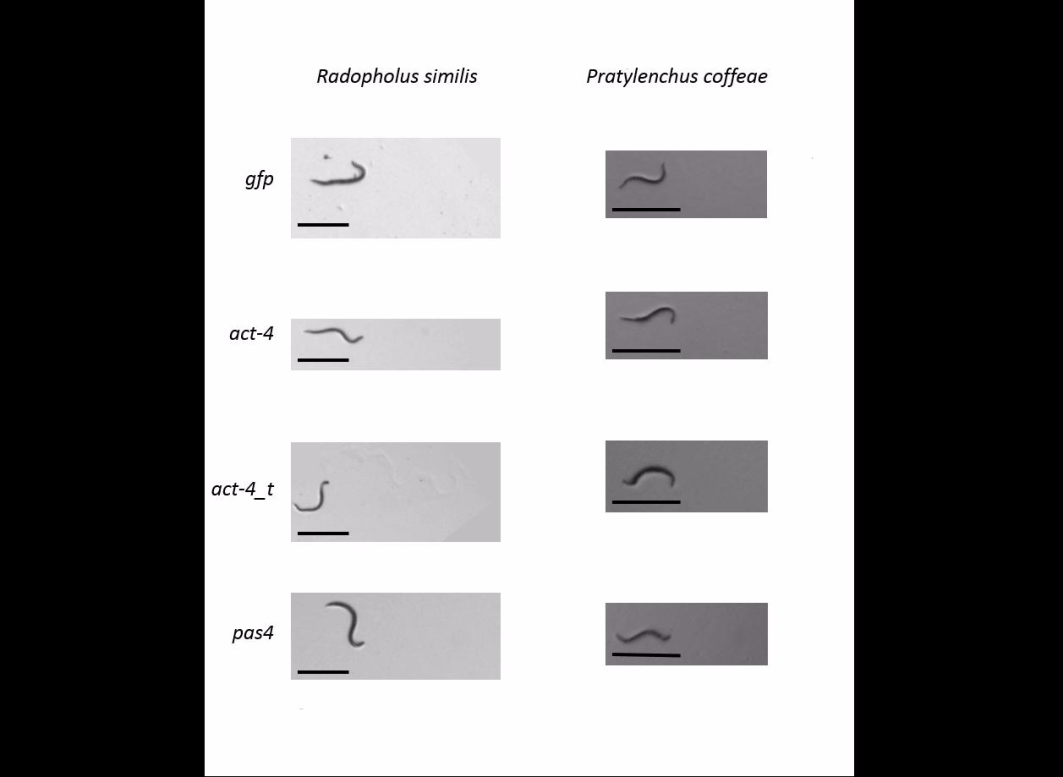

Supplement: Supplementary file 1 — Figure S1 Alignments of (a) act‐4 and (b) pas‐4 dsRNA molecules and their targets in the plant‐parasitic nematodes Radopholus similis, Pratylenchus coffeae, Helicotylenchus multicinctus and Meloidogyne incognita and the nontarget, Caenorhabditis elegans. Table S1 Primers used for dsRNA construct cloning. Linker restriction sites used for cloning are indicated in bold italics. Table S2 Primers used for transcript screening and quantification. Genes used as internal controls for normalising quantification indicated in bold. [file PBI-16-520-s001.docx]
